# Pressure injury flap surgery in people with spinal cord injury/disorder: surgical and rehabilitation outcomes

**DOI:** 10.1038/s41393-026-01226-w

**Published:** 2026-06-05

**Authors:** Evgeniya Zakharova-Luneva, Christy Hogan, Rachel Jones, Jo-anne Kemp, Darren Allon, Samuel Yang, Timothy Geraghty

**Affiliations:** 1 The Hopkins Centre, Griffith University, Brisbane, QLD Australia; 2 Spinal Injuries Unit, Queensland Spinal Cord Injuries Service, Department of Rehabilitation (DoR), Division of Allied Health and Rehabilitation (DAHR), Princess Alexandra Hospital, Metro South Health (MSH), Brisbane, QLD Australia; 3 Spinal Outreach Team, Queensland Spinal Cord Injuries Service, DoR, DAHR, Princess Alexandra Hospital, MSH, Brisbane, QLD Australia; 4 Department of Plastic and Reconstructive Surgery, Princess Alexandra Hospital, MSH, Brisbane, QLD Australia; 5 School of Medicine and Dentistry, Griffith University, Brisbane, QLD Australia

**Keywords:** Risk factors, Outcomes research

## Abstract

**Study design:**

Retrospective chart audit.

**Objectives:**

To describe the full range of complications in individuals with spinal cord injury or disorder (SCI/D) following flap repair of pelvic pressure injuries and explore factors associated with post-surgical complications.

**Setting:**

Tertiary hospital Spinal Injuries Unit.

**Methods:**

Data on 91 variables, including demographics, medical and surgical details, function, pre- and post-operative management, and complications were extracted for individuals admitted for flap surgery between 2015 and 2023. Descriptive and non-parametric analyses were performed.

**Results:**

Sixty-one individuals with SCI/D underwent 84 flap surgeries during the study period. At discharge 95% could sit for at least 4 h with 50% tolerating more than 8 h, however 15% (*n* = 9/59) had reduced ability to transfer and 27% (*n* = 17/63) needed increased personal care support. Being overweight (Y/N) was significantly associated with a 54% increase in total complications (B = 0.43, SE = 0.22, IRR = 1.54, 95% CI [1.01, 2.35], *p* = 0.045), however diabetes (Y/N) was not. Local complications were positively associated with number of pressure injuries at admission, previous flap surgeries, length of stay, days from surgery to sitting and discharge, osteomyelitis, spasticity on admission, and increased pain. General complications were associated with diabetes and days from surgery to discharge.

**Conclusions:**

Post-flap complications occur frequently, impacting rehabilitation progress and length of stay. By examining both local and general complications this study offers a broad-ranging view of the post-flap surgical and rehabilitation trajectory.

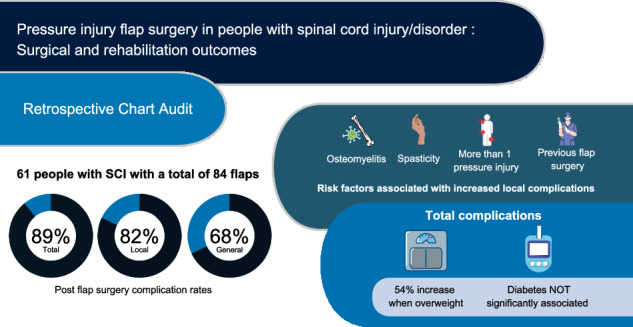

## Introduction

Pressure injuries (PIs) are increasingly prevalent secondary complications of spinal cord injury or disorder (SCI/D), profoundly affecting individuals’ quality of life with significant cost to the health system [1–3]. PIs are classified according to the National Pressure Injury Advisory Panel (NPIAP) staging system [4] with stage 1 and 2 often managed conservatively, whilst surgical management involving debridement and flap surgery is generally required for more complex stage 3 and 4 PIs [5].

Flap surgery for management of PIs often involves local axial-pattern flaps with a well-defined blood supply [6]. These are categorised as fasciocutaneous, which comprise skin, subcutaneous tissue and deep fascia; or myocutaneous flaps, involving the skin, subcutaneous and deep fascia, and underlying muscle [5, 6]. Documented complication rates following flap surgery vary between 21–74%, with complications often prolonging the length of stay (LoS) [7–12]. Local complications include wound dehiscence, infection, necrosis, haemorrhage, seroma, and sepsis from local infection [7, 9, 11–13]. Existing research has focused on the surgical wound which may overlook more general complications that influence post-surgical recovery and wound healing, including anaemia, sepsis, pneumonia, or new hospital-acquired PIs [9]. Whilst some previous research examines factors such as multidisciplinary (MD) postoperative management, risk screening, nutritional optimisation, spasticity management, and education, there is little evidence supporting the reduction in post-flap surgery complications associated with these strategies [5]. Very few studies have explored the risk factors that predispose individuals to develop such complications.

Meticulous, specialist surgical and rehabilitation management following flap surgery is crucial and whilst postoperative management protocols differ, they generally include a period of strict bed rest, between 2–8 weeks, followed by graded return to sitting and activities of daily living [5, 7, 9, 11]. Anecdotal evidence from the Spinal Injuries Unit (SIU) where this study was undertaken suggested concerning complication rates, with general complications often prolonging postoperative recovery, rehabilitation and LoS. Discharge delays are worrying and people with SCI/D describe the postoperative period as extremely “confining” due to prolonged LoS and social isolation, resulting in grief, depression and poorer adherence with treatment [5, 14]. Therefore, the current study aimed to: 1) comprehensively describe the range of local and general complications occurring in individuals with SCI/D following flap surgical repair for PI; and 2) examine potential factors contributing to post-surgery complications.

## Methods

### Study design and setting

**Fig. 1:**
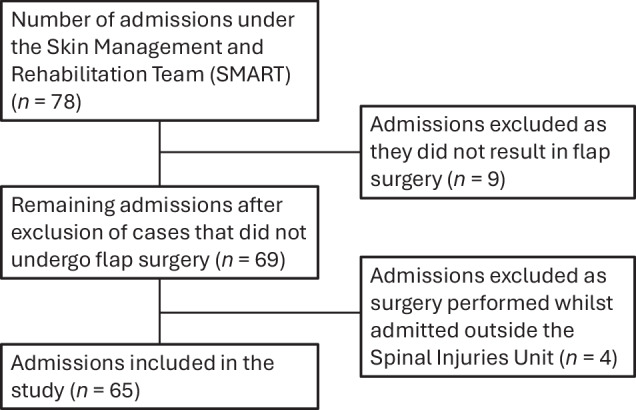
Flow chart of inclusion and exclusion of admissions.

This retrospective chart audit examined a consecutive cohort of adults (≥18 years) with SCI/D admitted to the SIU in Brisbane, Australia, for flap repair of pelvic PIs between January 2015 and December 2023. Individuals initially admitted to a different unit for flap surgery and subsequently transferred to the SIU were excluded due to postoperative protocol differences (Fig. 1). Ethical approval was granted by relevant public health (HREC/2024/QHFSS&PQ/109982) and university (GU: 2024/671) Human Research Ethics Committees. This research forms part of a larger mixed-methods study aiming to enhance the management of people with SCI/D and PIs needing flap-surgery.

The SIU is part of a publicly funded state-wide specialist SCI service [15], providing inpatient, outpatient and community services for individuals with SCI/D [16]. The SIU has 4 dedicated beds and an experienced MD Skin Management and Rehabilitation Team (SMART) to support people requiring an admission for flap surgery. The SMART includes a plastic surgeon specialised in flap surgery and a MD SCI/D team including a rehabilitation physician, nursing and allied health. All flap surgeries are performed by a single plastic surgeon with 11 years’ experience and expertise in flap surgery for people with SCI/D facilitating expert and consistent technical surgical skills and decision-making. Initial assessment is undertaken in the SIU SMART Clinic with pre-admission community management supervised by the Spinal Outreach Team [16]. Individuals appropriate for surgery are admitted directly to the SIU where they undergo both surgery and postoperative rehabilitation within the same facility.

### Surgical and postoperative management

Surgery was performed under general anaesthesia. The wound and sinus were debrided and covered using a fasciocutaneous or myocutaneous flap, depending on surgeon’s preference and clinical assessment. For individuals with multiple PIs, the decision to perform simultaneous or staged flap repairs was based on their ability to offload pressure simultaneously from both surgical sites postoperatively. One or two drains per flap were placed and remained for at least eight days.

Postoperative management involved appropriate positioning on an alternating air mattress and completely offloading the flap in side-lying or prone position for at least three weeks. Progress was monitored via weekly MD ward rounds and case conferences. When the surgical site was healed, gentle progressive hip ranging commenced, and lying on the flap for progressively longer periods was allowed. At four weeks, wheelchair sitting on a pressure redistribution air cushion was initiated and advanced gradually according to an established return to sitting regimen (Supplementary Material 1). Surgical techniques, peri- and postoperative protocols remained unchanged throughout the study period.

### Measures and procedure

Data were collected from medical records and included 91 variables (Supplementary Material 2), grouped into the following categories: demographics; medical and injury, aetiology of SCI/D and neurological classification, function at admission; preoperative management, surgical details; complications; and postoperative management. Neurological classification was reported according to the American Spinal Injury Association (ASIA)/ISCoS International Standards for Neurological Classification of Spinal Cord Injury (ISNCSCI) [17]. Osteomyelitis was recorded if prescription of antibiotics for this indication occurred in the perioperative period. Body mass index (BMI) adjusted for SCI/D was applied, with an ideal range defined as 16–22 kg/m²; values above this threshold were considered indicative of overweight or obesity [18]. At present, there are no established cut-off points to distinguish between overweight and obese categories within this population.

The primary outcome was complications following flap repair, occurring either before or after sitting commenced. Complications were further categorised into local and general complications, as previously described by Gelis et al. [9]. Local complications included wound infection or sepsis related to the surgical site, suture line dehiscence, haemorrhage, haematoma or seroma formation, partial flap necrosis or flap failure. Suture line dehiscence included any break in the surgical wound, including at the donor site. Wound infection was defined based on whether there was prescription of antibiotics (oral or intravenous) for this indication. General complications included systemic complications such as pneumonia, urinary tract infection (UTI) or urosepsis, or other non-flap related complications such as development of hospital-acquired PI. Severity of early postoperative complications (prior to sitting) were graded according to the Clavien-Dindo Classification with Grade I-II classed as minor complications, and Grade III-V as major complications [19].

### Statistical analyses

Data were collated using Microsoft Excel and analysed using IBM SPSS Statistics (Version 30, IBM, Armonk, NY). Descriptive analyses summarise demographic, injury and surgical information. Local, general, and total complication rates were calculated. Variables were examined for normality and outliers. Missing data were handled using pairwise deletion, allowing for maximum retention of the available data to preserve statistical power. To ensure clarity and transparency, the sample size (*n*) for each variable is reported alongside percentages throughout the results section. Missing data and valid percentages (i.e., percentages based on cases with available data) are reported in each table. Non-parametric Kendall’s tau-b correlations, accounting for numerous ties in count and binary data, examined associations between risk factors and other demographic and hospital-related variables of interest and number of complications. Due to data limitations planned linear regression analyses were not conducted, limiting our ability to assess the relationship between age and surgery wait time on complications. Instead, exploratory Mann-Whitney U analyses examined differences in the number of local, general, and total complications between those with and without risk factors previously known to impact complication rates, with at least 5 people in each group. Poisson regression was employed to examine the association between diabetes and being overweight and the count of total complications. To ensure independence of observations, inferential analyses were conducted using data from the most recent surgery for each participant (*n* = 61).

## Results

### Sample characteristics

Between 2015–2023, 61 people with SCI/D with a total of 121 PIs of varying stages underwent 84 flap repair surgeries across 65 admissions to the SIU. Most individuals were admitted for this purpose, however, two were originally admitted to the SIU for other reasons. Most individuals were male (*n* = 54/61; 89%) with a mean age of 53 (*n* = 84, *SD* = 13.7, range 27–74). Eighty-two percent (*n* = 50/61) had SCI/D of traumatic aetiology, 6.6% spina bifida (*n* = 4/61), and the remainder other non-traumatic causes (*n* = 7/61). Fifty-six percent had paraplegia (*n* = 34/61) and 67% had a complete injury (ASIA Impairment Scale (AIS) A; *n* = 41/61). Fifty percent had one PI per admission (*n* = 33/65; range 1–6). Ischial tuberosity (*n* = 58/120; 48%) was the most common PI location, followed by the greater trochanter (*n* = 20/120; 20%), sacrum (*n* = 20/120; 20%), and foot (*n* = 20/120; 20%). See Table 1 for demographics and injury data.

**Table 1:** Descriptive statistics of demographic and injury information for flap surgery participants.

Variable	N	Frequency	Valid %	*M* (*SD*)	Range
Age at admission (years)	61			52.1 (13.4)	27–74
Sex	61				
Male		54	88.5		
Female		7	11.5		
Marital Status	61				
Married/Partnered		18	29.5		
Single/Divorced/Widowed		43	70.5		
Geographical Location^a^	61				
Major City		40	65.6		
Regional		20	32.8		
Remote		1	1.6		
Aboriginal and/or Torres Strait Islander	61	6	9.8		
Pre-admission Employment (per admission)	64				
Employed/study		8	12.5		
Unemployed/on leave		49	76.6		
Retired		7	10.9		
Missing data	1				
Number of admissions	61	65		1.0 (1.0–1.0)^*†*^	1–3
1		58	95.1		
2		2	3.3		
3		1	1.6		
Time from injury to surgery (years)	82			22.6 (13.5)	2–48
Missing data	2				
SCI Cause	61				
Trauma		50	82.0		
Non-trauma		11	18.0		
Type of SCI/D^b^	61				
Paraplegia Complete (A)		27	44.3		
Paraplegia Incomplete (B-D)		7	11.5		
Tetraplegia Complete (A)		14	23.0		
Tetraplegia Incomplete (B-D)		9	14.8		
Spina Bifida		4	6.6		
Number of PIs^c^ (per admission)	65			1.0 (1.0–2.5)^*†*^	1–6
1		33	50.8		
2		16	24.6		
3		10	15.4		
4–6		6	9.2		
PI location	120				
Ischial Tuberosity (incl. perineum)		58	48.3		
Greater Trochanter		20	16.7		
Sacrum (incl. coccygeal)		20	16.7		
Foot (incl. ankle, malleolus, heel, toe, calcaneal)		20	16.7		
Other (i.e., perianal, elbow)		2	1.7		
Missing data	1				
PI stage	112				
1		6	5.4		
2		10	8.9		
3		11	9.8		
4		67	59.8		
Unstageable		13	11.6		
Healed		3	2.7		
SDTI^d^		2	1.8		
Missing	9				
PI size (cm^2^)	71			4.0 (2.0–16.0)^*†*^	0.20–400
Missing data	20				
Osteomyelitis	65	8	12.3		

^a^Geographical Location based on Australian Statistical Geography Standard (ASGS) Edition 3 (Australian Bureau of Statistics).

^b^SCI level and degree of impairment based on ASIA Impairment Scale.

^c^*PI* pressure injury.

^d^*SDTI* suspected deep tissue injury.

^†^Median (IQR).

### Flap repair surgery

Surgery details are displayed in Table 2. Median number of days from flap surgery to discharge was 123 (*n* = 84, IQR = 88.3–178.8, range 43–379). Most admissions involved one flap-surgery (*n* = 45/61; 75%); however, four individuals required multiple flap surgeries in a single admission (range 3–4). All except one flap were completed as single-stage surgery. Posterior thigh (*n* = 34/84; 40%) was the most common flap type and over two-thirds (*n* = 61/84; 73%) of flaps were fasciocutaneous.

**Table 2:** Descriptive statistics displaying surgical information.

Variable	N	Frequency	Valid %	*Median* (*IQR*)	Range
Number of flap surgeries	61	84		1.0 (1.0–1.5)	1–4
1		45	73.8		
2		12	19.7		
3		1	1.6		
4		3	4.9		
Type of Flap	84				
Posterior thigh		34	40.5		
Tensor fascia latae		14	16.7		
Z-plasty		11	13.1		
Axial pattern fasciocutaneous		2	2.4		
Random Pattern Rhombic		5	6.0		
Other fasciocutaneous		9	10.7		
Gracilis		6	7.1		
Buttock gluteal rotation		2	2.4		
Adductor magnus		1	1.2		
Tissue composition	84				
Fasciocutaneous		61	72.6		
Myocutaneous		23	27.4		
Days from admission to discharge	84			148.5 (108.5–250.8)	50–406
Days from flap surgery to discharge	84			123 (88.3–178.8)	43–379
Days from SMART^a^ clinic to surgery	64			413 (189.8–716.5)	13–1525

^a^*SMART* skin management and rehabilitation team.

### Functional status and equipment

At discharge, reduced ability with transferring was found for 15% of individuals (*n* = 9/59) and increased levels of personal care support were required for 27% (*n* = 17/63). Supplementary Table 1 outlines pre-admission and discharge functional status for the 65 admissions. For 95% (*n* = 54/57) sitting for at least 4 h was achieved by discharge, and in 47% (*n* = 27/57) sitting for at least 8 h was possible versus 55% (*n* = 26/47) and 17% (*n* = 8/47) at pre-admission, respectively. Prior to admission, 72% had a contracture (*n* = 47/65), 63% documented kyphoscoliosis and/or pelvic obliquity (*n* = 29/65), and 15% spasticity interfering with function (*n* = 10/65). Changes to existing equipment, predominantly bathing/toileting aids and wheelchairs was required in 51% (*n* = 43/84) of cases and 68% needed at least one new piece of equipment (*n* = 57/84), mostly toileting/bath aids and wheelchair cushions (see Supplementary Table 2).

### Co-morbidities and pre-admission management of PI

According to the BMI adjusted for SCI/D [18], 74% (*n* = 45/62) of people were overweight or obese at the time of surgery. Diabetes was present in 28% of the cohort (*n* = 18/65), whilst HbA1c was above 6.5% in 43% (*n* = 15/35) of those with available data. Twenty-two percent (*n* = 14/65) had undergone previous flap surgery.

General practitioners (*n* = 64/65; 98%), nurses (*n* = 61/65; 94%) and physiotherapists (*n* = 57/65; 88%) were common disciplines seen prior to surgery. The majority were reviewed by a SCI/D specialist via SIU General Clinic (*n* = 49/65; 75%), SMART Clinic (*n* = 47/65; 72%), or the Spinal Outreach Team (*n* = 77/84; 92%). See Supplementary Tables 3 and 4 for pre-surgery health professional involvement and descriptive statistics for comorbidities/blood biomarker levels.

### Complications and surgical revisions

**Fig. 2:**
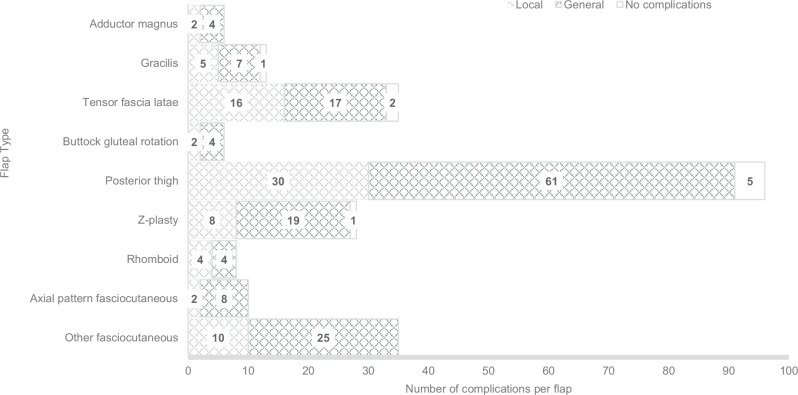
Number of local and general complications per flap type.

**Fig. 3:**
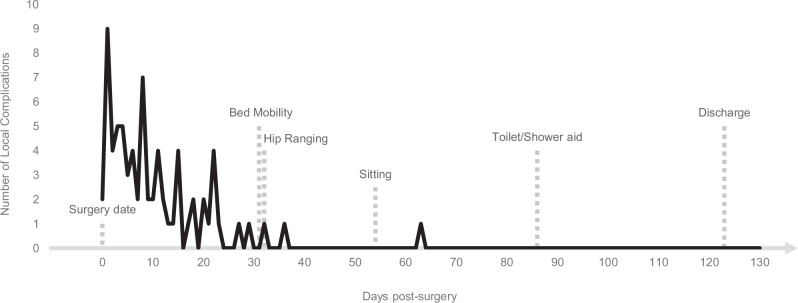
Frequency of local complications per day post-surgery. Dotted bars represent the median number of days to mobility milestones and discharge.

Across 84 flap surgeries, there were 230 complications (*Median* = 2.00, *IQR* = 1.00–4.00, range 0–9) with 165 occurring before and 65 after sitting commenced. These were further categorised into 79 local complications (*M* = 0.94, *SD* = 0.57, range 0–3) and 151 general complications (*Median* = 1.00, *IQR* = 0.00–3.00, range 0–7). Figure 2 displays complications by surgical flap type. Most pre-sitting complications were minor, Grade I or II (*n* = 57/75; 76%). Common local complications included suture line dehiscence and infection. UTI and hospital-acquired PI were the most common general complications. In contrast to general complications, local complications mostly occurred before sitting commenced (Table 3, Fig. 3).

**Table 3:** Frequencies and range for local and general complication information (*n* = 84).

Variable	Surgeries with complications (Valid %)	Prior to sitting Frequency (Valid %)	After sitting Frequency (Valid %)	Sum	Range
Clavien-Dindo Classification^a^ (*n* = 75)					
I	21 (28.0)				
II	36 (48.0)				
IIIa	4 (5.3)				
IIIb	12 (16.0)				
IVa	2 (2.7)				
V	0 (0)				
Missing = 9					
Local complication events (*n* = 84)	69 (82.1)	78 (98.7)	1 (1.3)	79 (Missing = 5)	0–3
0	15 (17.9)				
1	60 (71.4)				
2	8 (9.5)				
3	1 (1.2)				
Local complication type (*n* = 84)					
Infection req. oral antibiotics	10 (11.9)			10	0–1
Infection req. IV^b^ antibiotics	29 (34.5)			29	0–1
Sepsis	4 (4.8)			4	0–1
Collection req. drainage	4 (4.8)			4	0–1
Haematoma/seroma or bleeding	13 (15.5)			13	0–1
Suture line dehiscence req. irrigation	40 (47.6)			41	0–2
Suture line dehiscence not req. irrigation	19 (22.6)			19	0–1
Partial flap necrosis	2 (2.4)			2	0–1
Flap failure	1 (1.2)			1	0–1
Other local complication	5 (6.0)			6	0–2
Total General complications (*n* = 84)	57 (67.9)	87 (57.6)	64 (42.4)	151	0–7
0	27 (32.1)				
1	18 (21.4)				
2	13 (15.5)				
3	10 (11.9)				
≥4	16 (19.0)				
General complication type (*n* = 84)					
Pneumonia	6 (7.1)	6 (85.7)	1 (14.3)	7	0–2
Other respiratory	6 (7.1)	3 (50.0)	3 (50.0)	6	0–1
UTI/Urosepsis	35 (41.7)	27 (52.9)	24 (47.1)	51	0–5
Hospital-acquired PI^c^	21 (25.0)	16 (48.5)	17 (51.5)	33	0–4
Other skin issues	21 (25.0)	15 (65.2)	8 (34.8)	23	0–2
Gastrointestinal	7 (8.3)	5 (71.4)	2 (28.6)	7	0–1
Cardiovascular	5 (6.0)	1 (20.0)	4 (80.0)	5	0–1
Other complications^d^	17 (20.2)	13 (65.0)	7 (35.0)	20	0–2

^a^Grade I = Any deviation from the normal postoperative course without the need for pharmacological treatment or surgical, endoscopic and radiological interventions; Grade II = Requiring pharmacological treatment with drugs other than those allowed for grade I complications; Grade III = Requiring surgical, endoscopic or radiological intervention; Grade IIIa = Intervention not under general anaesthesia, IIIb = Intervention under general anaesthesia; Grade IV = Life-threatening complication requiring IC / ICU-management; IVa = single organ dysfunction, IVb = multiorgan dysfunction; Grade V = Death [19].

^b^IV = Intravenous.

^c^Pressure injury.

^d^Other complications included: acute kidney injury, reduced level of consciousness due to overdose, minimal trauma fracture, haematuria, post-operative anaemia etc.

A new hospital-acquired PI occurred following 25% (*n* = 21/84) of surgeries with 33 PIs in total (range 0–4; Table 3). Nearly two-thirds of new PIs were stage 1 or 2 (*n* = 21/33; 63%), but staging data was missing for 21% (*n* = 7/33; Supplementary Table 5). One-third occurred over the greater trochanter (*n* = 11/33; 33%). The majority (*n* = 14/33; 42%) were assessed as being related to strict post-surgery bed rest requirements. Twelve flap surgeries (*n* = 12/84; 14%) required surgical revision predominantly before sitting commenced (*n* = 11/14, 79%; after sitting *n* = 3/14, 21%; see Table 4).

**Table 4:** Frequencies of surgical revisions and other complicating factors during admission.

Variable	*n*	Frequency (valid %)	Total episodes (range)
Surgical revision / intervention^a^	84	12 (14.3)	14 (0–2)
0		72 (85.7)	
1		10 (11.9)	
2		2 (2.4)	
Type of revision / intervention	14		
Debridement		2 (14.3)	
Flap repair		3 (21.4)	
New flap		4 (28.6)	
Other^a^		5 (35.7)	
Other complicating factors			
Autonomic dysfunction^b^	84	17 (20.2)	55 (0–11)
Increased spasticity	84	12 (14.3)	
Increased pain	84	35 (41.7)	
Additional procedures^c^	84	13 (15.5)	17 (0–3)
Prior to sitting	17	4 (23.5)	
After sitting	17	13 (76.5)	
0 procedures		71 (84.5)	
1 procedure		10 (11.9)	
2–3 procedures		3 (3.6)	
Complicating factors during rehabilitation phase			
Inability to maintain optimal positioning in bed	84	12 (14.3)	
Due to:	12		
Pain / discomfort		7 (58.3)	
Psychological		1 (8.3)	
Spasticity		3 (25.0)	
Reduced function in bed		2 (16.7)	
Other		2 (16.7)	
Directly related to complication development	12	7 (58.3)	
Inability to adhere to sitting regime	84	11 (31.1)	
Due to:	11		
Pain / discomfort		5 (45.5)	
Psychological		8 (72.7)	
Reduced function in bed		4 (36.4)	
Cultural		4 (36.4)	
Began sitting before medical clearance		4 (36.4)	
Other		5 (45.5)	
Directly related to complication development	10	2 (20.0)	
Bed issues, malfunction or breakage	84	2 (2.4)	
Directly related to complication development	2	2 (100)	
Incontinence impacting on flap surgery site	84		
Urinary		16 (19.0)	
Faecal		3 (3.6)	
Directly related to complication development	17	0 (0)	
Return to bed rest episodes	84	34 (40.5)	57 (1–6)
1		22 (64.7)	
2		6 (17.6)	
3		3 (8.8)	
4		2 (5.9)	
6		1 (2.9)	
Reason for return to bed rest	57		
Suture line breakdown		35 (61.4)	
New PI^d^		16 (28.1)	
Other		6 (10.5)	

^a^Other surgical interventions included irrigation/washout, bone biopsy, Girdlestone’s procedure, and clipping of bleeding artery.

^b^Autonomic dysfunction post-surgery requiring medications or medical attendance.

^c^Examples of additional procedures included cystoscopy, intravesical Botulinum toxin, management of bladder/renal stones, gastroscopy etc.

^d^Pressure injury.

### Postoperative complicating factors

Increased pain was the most common complicating factor after flap surgery (*n* = 35/84; 42%) followed by autonomic dysfunction (*n* = 17/84; 20%). Furthermore, pain/discomfort was the most common factor related to inability to maintain optimal positioning in bed and the second most common factor related to reduced adherence to the sitting regimen. Psychological factors, often related to challenges tolerating the prolonged bed rest and LoS, was the predominant reason individuals were unable to adhere to the sitting regimen. Episodes of return to bed rest occurred after 41% (*n* = 24/84) of surgeries with 57 episodes in total (*Median* = 1.00, IQR = 1.00–2.00), with a median of 13.5 days (*n* = 34, IQR = 5.0–22.3), mostly due to suture line breakdown and development of a new PI (Table 4).

### Postoperative bed rest

Duration of strict postoperative bed rest varied across the cohort. The median number of days from surgery to commencement of bed mobility was 31 (*n* = 79, *IQR* = 22.0–50.0, range 9–218), to commencement of sitting was 54 (*n* = 82, IQR = 35.8–84.0, range 23–240), and to commencement of sitting on a toilet/bath aid was 86 days (*n* = 73, IQR = 59.0–136.5, range 37–310). Figure 3 displays median days to key postoperative milestones and occurrence of local complications.

### Correlational analyses

Kendall’s tau-b correlational analyses were conducted to examine associations between variables of interest and complication rates. There were significant positive associations between being overweight (τ_b_ = 0.273, *p* = 0.018), length of stay (τ_b_ = 0.309, *p* < 0.001), days from surgery to discharge (τ_b_ = 0.361, *p* < 0.001), days from surgery to sitting (τ_b_ = 0.325, *p* < 0.001), diabetes mellitus (τ_b_ = 0.226, *p* = 0.045), urinary incontinence impacting on the flap site (τ_b_ = 0.232, *p* = 0.040), with total number of complications post-flap surgery. Correlations for sub-analyses (local and general complications) are displayed in Table 5, with positive associations found between local complications and number of PIs on admission, history of previous flap surgeries, LoS, days from surgery to discharge, days from surgery to sitting, osteomyelitis, spasticity on admission, and increased pain. General complications were positively associated with being overweight, days from surgery to discharge, and diabetes.

**Table 5:** Kendall’s tau-b correlational analyses between demographic and hospital-related variables of interest and number of complications (*n* = 61).

Variable	*n*	Local complications	General Complications	Total complications
Age	61	−0.058	0.162	0.113
Overweight (binary Y/N)	58	0.078	0.265* (*p* = 0.024)	0.273* (*p* = 0.018)
More than 1 PI at admission (binary Y/N)	61	0.485*** (*p* < 0.001)	−0.003	0.140
History of previous flap surgery (binary Y/N)	61	0.356** (*p* = 0.004)	−0.136	−0.014
Days from referral to surgery	56	0.163	−0.084	−0.014
Days from SMART^a^ clinic to surgery	47	0.110	0.050	0.068
Number of hospital admission days for the same pressure injury	32	−0.076	−0.163	−0.164
Length of stay (admission to discharge)	61	0.456*** (*p* < 0.001)	0.182	0.309*** (*p* < 0.001)
Days from surgery to discharge	61	0.416*** (*p* < 0.001)	0.255** (*p* = 0.007)	0.361*** (*p* < 0.001)
Days from surgery to sitting	61	0.478*** (*p* < 0.001)	0.178	0.325*** (*p* < 0.001)
Osteomyelitis	58	0.305* (*p* = 0.013)	−0.090	0.040
Tetraplegia (vs paraplegia/spina bifida)	61	−0.012	0.049	0.049
Myocutaneous flap	61	0.205	−0.030	0.003
Diabetes mellitus (Y/N)	61	−0.020	0.257* (*p* = 0.025)	0.226* (*p* = 0.045)
Current smoker	61	−0.019	−0.198	−0.197
Spasticity interfering with positioning or transfers on admission	61	0.271* (*p* = 0.028)	0.162	0.195
Kyphoscoliosis/pelvic obliquity	42	−0.078	0.247	0.215
Urinary incontinence prior to admission	61	0.177	0.054	0.121
Bowel incontinence prior to admission	61	−0.041	−0.182	−0.185
Urinary incontinence impacting on flap site	61	0.183	0.153	0.232* (*p* = 0.040)
Faecal incontinence impacting on flap site	61	0.170	−0.090	0.028
Increased pain	61	0.337** (*p* = 0.006)	0.008	0.114
Mental health	61	0.036	−0.097	−0.038
Traumatic Brain Injury	61	0.067	−0.113	−0.054

^a^*SMART* skin management and rehabilitation team.

*p < 0.05, **p < 0.01, ***p < 0.001.

### Inferential analyses

Mann-Whitney U results are displayed in Supplementary Table 6 and indicated that individuals with osteomyelitis (*p* = 0.019, *r* = −0.32, medium), spasticity on admission (*p* = 0.038, *r* = −0.28, small), more than 1 PI (*p* < 0.001, *r* = −0.50, large), increased pain following surgery (*p* = 0.006, *r* = −0.35, medium), and a previous flap surgery (*p* = 0.004, *r* = −0.37, medium) had significantly higher local flap complications compared to those without these risk factors. Individuals with urinary incontinence were likely to have significantly higher total complications compared to those without (*p* = 0.040, *r* = −0.26, small). No other significant differences were found.

### Regression analyses

A Poisson regression was performed to examine the relationship between number of total complications and two risk factors, namely diabetes mellitus and being overweight (adjusted SCI categories). The inclusion of covariates significantly improved model fit compared to the intercept-only model, LR *Χ*^2^ (2) = 9.53, *p* = 0.009. Being overweight was associated with a 54% increase in predicted rates of total complications (B = 0.43, SE = 0.22, IRR = 1.54, 95% CI [1.01, 2.35], *p* = 0.045), however, diabetes mellitus was not (B = 0.22, SE = 0.17, IRR = 1.25, 95% CI [0.90, 1.72, *p* = 0.187).

## Discussion

This study highlights the challenges in comprehensive management for individuals with SCI/D and complex PIs needing surgical flap repair, with many people (89%) experiencing postoperative complications, even in a highly specialised and experienced SCI service. Reassuringly, most local complications prior to sitting were minor in severity and 95% of admissions resulted in the ability to sit for at least four hours at discharge with about half sitting for more than eight hours. This is a substantial improvement on pre-admission sitting time and indicative of an overall positive outcome.

Overall, higher BMI significantly increased an individual’s total complication rate by 54%, consistent with findings from previous studies [7, 10]. These findings highlight the importance of weight optimisation both preoperatively and during prolonged periods of bed rest, when reduced physical activity and suboptimal nutrition may contribute to weight gain and increase the risk of complications. The impact of postoperative weight gain, as well as optimal preventive management strategies, warrants further investigation. In contrast, diabetes was not identified as a risk factor for total complications in our sample, in keeping with prior findings [7, 9, 12, 13]. However, given the association between diabetes and wound infection, larger studies are needed to confirm a predictive relationship between diabetes and overall complication rates [12].

Complications being associated with a prolonged post-surgery hospital stay, median of 123 days, is consistent with previous research [10, 13]. Local wound complications, despite often being classified as minor complications according to the Clavien-Dindo Classification, had a significant impact on recovery, frequently delaying progression through early postoperative milestones, including resuming bed mobility and wheelchair sitting. Slowing of the post-surgical rehabilitation pathway was required when complications occurred after sitting had commenced, further extending hospitalisation. Rehabilitation was further complicated by the presence of multiple comorbidities with 16% of people undergoing interventions unrelated to their flap surgery, and by contributing factors including pain, spasticity and urinary incontinence impacting the surgical site that increased the rate of postoperative complications.

Previous studies often focused on local complications, reporting wide variability in complication rates, between 21–74%, which may reflect the absence of standardised definitions for local complications [7–9, 11, 12, 20]. The local complication rate in this study of 82% is higher than previously reported but may be a consequence of the aim to report complications as comprehensively as possible. In contrast, previous studies have often excluded less severe complications, delayed wound healing, and complications occurring after sitting and in non-compliant individuals [7, 9, 21]. Some postoperative protocols entail mobilisation after discharge in the community, likely leading to underreporting of minor complications [11, 22].

Notably, most flaps utilised in this cohort were posterior thigh flaps. Fasciocutaneous flaps have previously been associated with higher rates of wound dehiscence but lower rates of infection [23]. In the present study, however, both wound dehiscence (70%) and local flap infection (46%) rates were high compared to previous studies (31–63% and 6.5–27%, respectively) [7–9, 12] and may reflect differences in patient characteristics, surgical technique, or, more likely, the broader and more inclusive definitions of complications applied in this study.

Major complications were less common compared to previous research with a combined haematoma, seroma or bleeding rate of 16% compared to 19.5–26.5%, a 2.4% rate of partial necrosis versus 6.8–26.9%, and 1.2% flap failure rate versus 4.2–21.2% [7–9, 24]. It may be that, in the current cohort a more concerted approach to managing minor complications early, (e.g., returning to bed rest), prevented major complications, while potentially extending the postoperative period and LoS. Osteomyelitis being associated with increased risk of local flap complications is also consistent with previous findings [12, 13]. Given the diagnostic challenge with osteomyelitis and potential differences in management and outcomes of acute versus chronic presentations, further research is required to better inform clinical practice [25, 26]. Local flap complications were more likely in individuals with previous flap surgeries or multiple PIs at admission. This is expected clinically, as navigating existing scar tissue can make flap surgery more technically challenging, and managing concurrent PIs adds complexity to postoperative positioning.

To our knowledge, this is the first study to comprehensively document general complications in this population. General complications occurred in 68% of cases both before and after sitting commenced with UTIs, hospital-acquired PIs, skin issues such as dermatitis, and pneumonia most common. The occurrence of any new PIs is concerning, however 64% were stage 1 or 2 only and able to be managed conservatively. Most occurred over the greater trochanter during the strict postoperative bed rest period which poses a major additional challenge to management, as flap healing will likely be prioritised over offloading of the new PI. Other complicating factors including reduced adherence to the postoperative bed rest protocol, systemic illness, and equipment failure may have contributed to the development of complications but should be considered preventable through comprehensive MD preoperative assessment and optimisation [10].

While it is well-known that there is a subgroup of individuals undergoing flap repair who are unable to maintain the strict adherence to post-flap bed rest and mobilisation protocols required, limited research focuses on this group. The current study found inability to maintain bed positioning in 14% of cases, with pain being the primary reason (58%) and spasticity, limited function, and psychological factors also contributing. These complicating factors were clinically associated with new complications in over half of cases. Fewer people had difficulty adhering to the progressive sitting protocol, with psychological factors and pain being the primary reasons. However, complications related to non-observance of the sitting protocol were seen in only 20%, suggesting that the initial bed rest period may be more critical for wound healing. These findings are consistent with previous research emphasising the demanding physical and psychological nature of the postoperative stage, with long periods of strict bed rest resulting in a range of psychological complications including depression and reduced compliance [27, 28]. Lived experience research describes body image changes, pain, loss of privacy and control, boredom, frustration, depression, social isolation, and grief during this period [3, 14, 29]. This highlights the critical role of comprehensive psychosocial and pain assessment and management in both preoperative and postoperative periods [5, 10].

Previous studies reported median healing times of 46–48 days, with only 70% of wounds healed at three months, indicating that prolonged bed rest may be necessary following flap surgery [9, 20]. In the current study 41% of cases required an episode of return to bed rest after sitting had commenced for a median of 13.5 days, primarily due to concerns regarding skin integrity. This illustrates the complexity of postoperative recovery and warrants further investigation into strategies that would promote better wound healing.

A substantial proportion of people required either new equipment or modifications to existing equipment. This suggests that the original equipment was inappropriate or that equipment needs changed following flap surgery, possibly due to deconditioning or muscle wasting from prolonged immobilisation or location of new scar lines [30]. This is consistent with the additional finding that in 15% of cases, the method of transferring was downgraded (e.g., from horizontal transfers to requiring a hoist) at discharge. It is possible that, for a sub-group of people undergoing this complex surgery, it is not possible to return to previous functional levels by discharge, despite best efforts by the individual and treating team. However, it may also be that with a longer or more intense period of postoperative rehabilitation these functional losses could be ameliorated. Preoperative counselling and preparation should inform individuals with SCI/D undergoing flap surgery and their family and carers, as well as their support organisations and funding bodies, of the potential for postoperative functional decline, which may be temporary or permanent, and the possible requirement for ongoing rehabilitation, increased community support and associated additional funding on discharge.

### Limitations

This study is limited by its small size and retrospective, single-surgeon, single-centre design. The limited cohort reduced statistical power limiting our ability to detect small effects and non-normality of data prohibited meaningful analysis of potential risk factors above those included in the Poisson regression. Therefore, results need to be interpreted with caution. Additionally, missing data inherent to retrospective collection may have affected the accuracy of the results. As the data were derived from a single centre, findings may not be generalisable to other settings or populations.

## Conclusion

This study underscores the high rate and multifactorial nature of postoperative complications following flap repair in individuals with SCI/D and the substantial challenges faced during recovery. By capturing both local and general complications this study offers a comprehensive view of the post-flap surgical and rehabilitation trajectory. Despite the challenges, most people achieved favourable sitting outcomes, illustrating the potential for meaningful recovery with appropriate management. Higher BMI was associated with increased overall complication rates and should be considered in both pre- and postoperative management. Diabetes did not increase the risk of complications in this cohort, however future studies with larger samples may yet demonstrate a significant association. The findings will inform the development of a revised clinical pathway for this population at the study site. The study reinforces the need for holistic, MD assessment and management in specialised centres to minimise complications and optimise outcomes, an approach increasingly supported by emerging evidence [10, 11, 31]. Greater attention to early detection and management of osteomyelitis, pre-operative preparation and post-discharge support, optimisation of the management of pain, spasticity and continence, and provision of comprehensive psychosocial support throughout the process is strongly recommended. Further research is needed to clarify risk factors, better understand the impact of pain, psychosocial, functional, and other rehabilitation-related considerations in post-surgical planning, and develop targeted strategies to reduce complications, LoS, and support optimal rehabilitation outcomes.

## Supplementary information

Supplementary material

## Data Availability

Data analysed in this study is available from the corresponding author upon reasonable request.
