# Supplementary material for: Pressure injury flap surgery in people with spinal cord injury/disorder: surgical and rehabilitation outcomes
Source: Spinal Cord. 2026 Jun 5;64(8):716–26. doi: 10.1038/s41393-026-01226-w (PMC13441881; doi:10.1038/s41393-026-01226-w)
Supplement: Supplementary file 1 [file 41393_2026_1226_MOESM1_ESM.docx]

**Supplementary material Legend**

**Supplementary material 1:** Post-operative protocol for post-flap repair used at the study site including bed rest and post-operative management, and return to mobility and sitting regimen.

**Supplementary material 2:** List detailing all 91 variables collected via the chart audit covering the following domains - demographics, pre-operative management of PI, function at admission, medical and injury data, surgical details, complications, and post-operative management.

**Supplementary Table 1:** Table displaying the frequencies and percentages for participant functional status prior to admission and at discharge for the 65 admissions.

**Supplementary Table 2:** Table displaying the frequency and percentage of health professional involvement prior to surgery.

**Supplementary Table 3:** Table displaying descriptive statistics for comorbidities and blood biomarker levels documented for each of the 65 admissions.

**Supplementary Table 4:** Table displaying the frequency and percentage of PIs acquired post flap surgery during hospital admission. PI stage, location, and cause are also displayed.

**Supplementary Table 5:** Table displaying the frequency and percentage of new equipment and changes required to existing equipment post-flap surgery.

**Supplementary Table 6:** Table displaying Mann-Whitney U results, examining the difference in the number of local, general and total complications between those with and without risk factors, previously known to impact complication rates.

**Supplementary material 1**

**Pressure injury post-flap repair protocol Queensland Spinal Cord Injuries Service**

**Weeks 0-3 post flap repair**

- Strict bed rest and offloading in side-lying (on opposite side to the operated flap) or prone positioning on appropriate alternating air mattress
- Regular wound monitoring and twice daily betadine paint to suture line
- Pain management
- Nutritional support provided by dietician
- Psychosocial support provided by psychologist and social worker
- Bladder management:
  - insertion of indwelling catheter preoperatively if previously voiding *or*

managing bladder via clean intermittent self-catheterisation

- continue until sufficiently mobile to resume previous bladder management
- Bowel management:
  - bowel elimination in bed
- Sponge in bed whilst sutures in situ
- Surgical drains removed day 8, or as clinically appropriate, guided by plastic surgeon
- Sutures removed between week 2-3, or as clinically appropriate, guided by plastic surgeon
- Once sutures removed:
  - cease betadine paint and moisturise wound at least twice daily
  - commence showering on shower trolley
  - transferring via Patslide and two slide sheets in side-lying with surgical site offloaded
- At week 3 or once wound healing assessed to be adequate:
  - commence hip ranging (aiming to achieve 90°) and
  - free bed mobility (laying supine and on side of flap with bed flat, starting with 1 hour at a time and building time up as clinically appropriate)

**Weeks 4+**

- At 4 weeks and/or once surgical wound fully healed, commence sitting regime per schedule below:

| **Day** | **Sitting time (morning)** | **Sitting time (afternoon)** |
| --- | --- | --- |
| 1 | - | 30 minutes |
| 2 | 30 minutes | 30 minutes |
| 3 | 45 minutes | 45 minutes |
| 4 | 1 hour | 1 hour |
| 5 | 1 hour | 1 hour |
| 6 | 1.5 hours | 1.5 hours |
| 7 | 2 hours | 2 hours |
| 8 | 2 hours | 2 hours |
| 9 | 2.5 hours | 2.5 hours |
| 10 | 3 hours | 3 hours |
| 11 | 3 hours | 3 hours |
| 12 | 3.5 hours | 3.5 hours |
| 13 | 3.5 hours | 3.5 hours |
| 14 | 4 hours | 4 hours |
| A minimum of 2 hours rest and offloading of the flap is advised between the two daily sits. Once 4 hours are reached, this becomes logistically challenging, and often the second sit is shorter.  After 4 hours, progress as tolerated, increasing by no more than 1-hourly increments daily. | | |

- The decision to commence wheelchair sitting is made by the multi-disciplinary team during a joint ward round:
  - wheelchair sitting is started on a pressure redistribution cushion, usually an air cushion, and transfers are usually done via hoist with choice of sling that avoids scar lines
  - The choice of cushion and transfer method is based on multi-disciplinary assessments, conducted prior and post flap surgery
- The skin is monitored closely and sitting schedule prolonged or paused at the discretion of the multi-disciplinary team if there is reddening or wound dehiscence
- After achieving a minimum 4 hours of wheelchair sitting, without compromise to the surgical wound:
  - gradual use of a toilet or bath aid is commenced (e.g., commode, shower chair, bench),
  - mattress downgrade (e.g., from air to foam mattress if clinically appropriate)
  - transfer practice if required
  - other wheelchair cushions can be trialled if clinically appropriate
  - *only one change is made each day i.e. sitting progression, toilet/bath aid progression, mattress change, commencement of transfer practice or trial of new wheelchair cushion*
- All equipment is reviewed and pressure mapping performed if indicated
- Shower or toilet bath aid trials begin with dry sitting, starting at approximately 15 minutes and gradually increasing as clinically appropriate to accommodate the bowel routine
  - once skin tolerance to dry sitting is established, a wet trial is initiated
- Patients are discharged home once:
  - they tolerate sufficiently long periods of wheelchair sitting to manage at home with available personal supports
  - have re-established bowel and bladder routines, and have appropriate equipment in place
  - care is transferred to community teams who support the patient on discharge

**Throughout the admission**

- Weekly multi-disciplinary ward rounds and discussion at case conference
- At least one Goal Planning Meeting involving the person post flap surgery and the multi-disciplinary team

**Supplemental material 2**

| **Full list of Data Variables (*n* = 91)** | |
| --- | --- |
| **Demographics (6 variables)**   - Age at admission - Sex - Marital status - Aboriginal and/or Torres Strait Islander status - Postcode of usual residence - Employment status | **Medical and Injury data (28 variables)**   - Years from injury to admission - Date of spinal cord injury - Age at initial spinal cord injury - ISNSCI classification - SCI lesion characteristics (tetraplegia / paraplegia, complete / incomplete) - Aetiology of SCI - Type of SCI (traumatic / non-traumatic) - Weight - Height - BMI - Smoking status - Presence of comorbidities:   Diabetes mellitus  Cardiovascular  Chronic kidney disease  Peripheral vascular disease  Mental health  Obstructive sleep apnoea  Other respiratory disorders  Traumatic brain injury  Previous flap surgeries  Previous pressure injuries  Other comorbidities   - Blood Biomarkers   Haemoglobin  Albumin  HbA1c  Folate  Vitamin B12  Iron studies  **Surgical details (8 variables)**   - Referral date - First Skin Management and Rehabilitation Team (SMART) clinic review - Number of pressure injuries at admission - Location of pressure injuries - Presence of underlying osteomyelitis - Number of flaps performed - Type of flap - Surgical date |
| **Pre-operative management of pressure injury (5 variables)**   - Medical and surgical input prior to surgery - Allied health input prior to surgery   Dietician  Physiotherapist  Occupational therapist  Psychologist  Social worker  Other   - Nursing input prior to surgery - Spinal Outreach Team involvement prior to surgery - Previous hospital admissions for the same pressure injury |  |
| **Function at admission (10 variables)**   - Mode of mobility - Mode of transfers - Independence with transfers - Independence with bed mobility - Number of hours wheelchair sitting prior to admission - Disability funding - Bowel management – mode and presence of incontinence - Bladder management – mode and presence of incontinence - Presence of spasticity which interferes with positioning or transfers - Contractures |  |
| **Complications (18 variables)**   - Intra-operative complications - Dates of post-operative complications - Local post-operative complications   Local infection managed with oral antibiotics  Local infection managed with intravenous antibiotics  Sepsis  Haematoma or bleeding  Suture line dehiscence requiring irrigation  Suture line dehiscence not requiring irrigation  Partial necrosis  Flap failure   - Surgical revision – type and date - General postoperative complications   Hospital-acquired pressure injury  Pneumonia  Urinary tract infection or urosepsis  Autonomic dysfunction  Spasticity  Pain  Other | **Post-operative management (16 variables)**   - Factors that affected positioning (if present) - Factors that affected adherence to sitting regime (if present) - Malfunction of equipment - Urinary incontinence - Faecal incontinence - Date commenced bed mobility - Date commenced hip ranging - Date commenced wheelchair sitting - Date commenced sitting on shower / bath aid - Number of episodes requiring return to bed rest - Prescription of new equipment - Modification of existing equipment - Hours sitting at discharge - Transfer method - Independence with transfers - Increase in personal care supports at discharge |

Supplementary Table 1. Frequency of pre-admission and discharge functional status for each admission (*n* = 65)

|  | **Pre-admission** | | | **Discharge** | | |
| --- | --- | --- | --- | --- | --- | --- |
| **Variable** | ***N*** | **Freq** | **Valid %** | ***N*** | **Freq** | **Valid %** |
| Mobility | 65 |  |  |  |  |  |
| Walking |  | 2 | 3.1 |  |  |  |
| Manual wheelchair |  | 47 | 72.3 |  |  |  |
| Power wheelchair (incl. power assist, scooter) |  | 31 | 47.7 |  |  |  |
| Transfers | 65 |  |  | 65 |  |  |
| Stand transfer |  | 3 | 4.6 |  | 2 | 3.1 |
| Horizontal without slideboard |  | 31 | 47.7 |  | 6 | 9.2 |
| Horizontal with slideboard |  | 12 | 18.5 |  | 27 | 41.5 |
| Hoist and sling |  | 30 | 46.2 |  | 35 | 53.8 |
| Patslide |  | 1 | 1.5 |  | 0 | 0 |
| Transfer independence | 65 |  |  | 64 |  |  |
| Independent |  | 35 | 53.8 |  | 24 | 37.5 |
| Assisted |  | 32 | 49.2 |  | 40 | 62.5 |
| Missing data |  |  |  | 1 |  |  |
| Bed Mobility | 64^◊^ |  |  |  |  |  |
| Assisted |  | 21 | 32.8 |  |  |  |
| Independent |  | 43 | 67.2 |  |  |  |
| Missing data | 1 |  |  |  |  |  |
| Hours sitting | 47^◊^ |  |  | 57 |  |  |
| 0 hours |  | 10 | 21.3 |  | 2 | 3.5 |
| >0 but <2hrs |  | 10 | 21.3 |  | 0 | 0 |
| ≥ 2 but <4 hrs |  | 1 | 2.1 |  | 1 | 1.8 |
| ≥4 but <6 hrs |  | 12 | 25.5 |  | 11 | 19.3 |
| ≥6 but <8hrs |  | 6 | 12.8 |  | 16 | 28.1 |
| ≥8hrs |  | 8 | 17.0 |  | 27 | 47.4 |
| Missing data | 18 |  |  | 8 |  |  |
| Bowel Management | 65 |  |  |  |  |  |
| Incontinent prior to admission |  | 10 | 15.4 |  |  |  |
| UMN^a^ bowel regime |  | 45 | 69.2 |  |  |  |
| LMN^b^ bowel regime |  | 6 | 9.2 |  |  |  |
| Transanal irrigation |  | 3 | 4.6 |  |  |  |
| Colostomy / ileostomy |  | 8 | 12.3 |  |  |  |
| MACE^c^ / Chait |  | 1 | 1.5 |  |  |  |
| Spontaneous |  | 3 | 4.6 |  |  |  |
| Urinary Management | 65 |  |  |  |  |  |
| Incontinent prior to admission |  | 15 | 23.1 |  |  |  |
| SPC^d^ |  | 24 | 36.9 |  |  |  |
| IDC^e^ |  | 12 | 18.5 |  |  |  |
| ICSC^f^ |  | 23 | 35.4 |  |  |  |
| Urinary diversion |  | 4 | 6.2 |  |  |  |
| Spontaneous voiding |  | 2 | 3.1 |  |  |  |
| Uridome |  | 3 | 4.6 |  |  |  |
| Reflex voiding |  | 3 | 4.6 |  |  |  |
| Spasticity interfering with positioning or transfers | 65 | 10 | 15.4 |  |  |  |
| Contractures | 65 | 47 | 72.3 |  |  |  |
| Ankle |  | 16 | 34.8 |  |  |  |
| Knee |  | 16 | 34.8 |  |  |  |
| Hip |  | 10 | 21.7 |  |  |  |
| Kyphoscoliosis / pelvic obliquity |  | 29 | 63.0 |  |  |  |

^◊^ Variable had some missing data; therefore, results are reflective of available data. ^a^UMN = upper motor neuron; ^b^LMN = upper motor neuron; ^c^MACE = Malone antegrade continence enema; ^d^SPC = suprapubic catheter; ^e^IDC = indwelling urinary catheter; ^f^ICSC = intermittent clean self-catheterisation.

Supplementary Table 2. Frequency of equipment revisions required after surgery

| **Variable** | ***n*** | **Frequency** | **%** |
| --- | --- | --- | --- |
| New equipment needed | 84 | 57 | 67.9 |
| Toilet / bath aid |  | 30 | 52.6 |
| Bed / mattress |  | 15 | 26.3 |
| Hoist / Sling |  | 16 | 28.1 |
| Wheelchair cushion |  | 28 | 49.1 |
| Wheelchair (MWC, PWC, power assist) |  | 18 | 31.6 |
| Wheelchair backrest |  | 2 | 3.5 |
| Other |  | 4 | 7.0 |
| Changes to existing equipment required | 84 | 43 | 51.2 |
| Bath / toilet aid |  | 23 | 53.5 |
| Wheelchair (manual/power) |  | 17 | 39.5 |
| Wheelchair Cushion |  | 8 | 18.6 |
| Wheelchair backrest |  | 14 | 32.6 |
| Other (chest strap, air adaptor pad) |  | 4 | 9.3 |

MWC = manual wheelchair, PWC = power-drive wheelchair

Supplementary Table 3. Frequency of health professional involvement prior to surgery for each admission

| **Variable** | **N** | **Frequency** | **%** |
| --- | --- | --- | --- |
| Allied health | 65 |  |  |
| Dietitian |  | 23 | 35.4 |
| Physiotherapist |  | 57 | 87.7 |
| Occupational Therapist |  | 52 | 80.0 |
| Psychologist |  | 4 | 6.2 |
| Social Worker |  | 35 | 53.8 |
| Other allied health |  | 1 | 1.54 |
| Nursing | 65 | 61 | 93.8 |
| Medical | 65 |  |  |
| General Practitioner |  | 64 | 98.5 |
| SIU^a^ General Clinic |  | 49 | 75.4 |
| SMART^b^ clinic |  | 47 | 72.3 |
| Other plastic surgery (private, general/inpatient) |  | 22 | 33.8 |
| Other surgical (orthopaedic/general) |  | 21 | 32.3 |
| Other medical (general rehabilitation/infectious disease) |  | 7 | 10.8 |
| SPOT^c^ involvement | 84 | 77 | 91.7 |
| Wound management advice |  | 74 | 96.1 |
| Equipment review / posture / seating |  | 74 | 96.1 |
| Review of transfers |  | 51 | 66.2 |
| Bowel/Bladder Management |  | 13 | 16.9 |
| Assistance with funding |  | 18 | 23.4 |
| Psychosocial support, adjustment and counselling |  | 8 | 10.4 |
| Other SPOT service |  | 11 | 14.3 |

*^a^SIU = Spinal Injuries Unit; ^b^SMART = Skin Management and Rehabilitation Team; ^c^SPOT = Spinal Outreach Team.*

Supplementary Table 4. Descriptive statistics for comorbidities and blood test results per admission

| **Variable** | **N** | **Frequency (Valid %)** | **M(SD)** | **Range** |
| --- | --- | --- | --- | --- |
| Total Comorbidities | 65 |  | 7.3 (2.9) | 1-15 |
| BMI ^a^ (at time of surgery) | 62^◊^ |  | 26.4 (6.6) | 11.9-41.0 |
| Underweight |  | 4 (6.5) |  |  |
| Healthy weight |  | 12 (19.4) |  |  |
| Overweight or obese |  | 46 (74.2) |  |  |
| Missing | 3 |  |  |  |
| Smoking | 65 |  |  |  |
| Never smoked |  | 29 (44.6) |  |  |
| Ex-smoker |  | 19 (29.2) |  |  |
| Current smoker |  | 17 (26.2) |  |  |
| Diabetes Mellitus | 65 | 18 (27.7) |  |  |
| Cardiovascular | 65 | 14 (21.5) |  |  |
| Obstructive Sleep Apnoea | 65 | 22 (33.8) |  |  |
| Other respiratory disorder | 65 | 11 (16.9) |  |  |
| Chronic Kidney Disease | 65 | 6 (9.2) |  |  |
| Mental health | 65 | 27 (41.5) |  |  |
| Traumatic brain injury | 65 | 11 (16.9) |  |  |
| Previous flap surgery prior to admission | 65 | 14 (21.5) |  |  |
| Number of previous flap surgeries | 14 |  | 2.0 (1.0-2.3)^†^ | 1-6 |
| 1 surgery |  | 5 (35.7) |  |  |
| 2 surgeries |  | 6 (42.9) |  |  |
| 3 surgeries |  | 2 (14.3) |  |  |
| 6 surgeries |  | 1 (7.1) |  |  |
| Number of other comorbidities | 65 |  | 3.6 (2.2) | 0-8 |
| Blood levels |  |  |  |  |
| Hb | 65 |  | 127.8 (17.4) | 81-163 |
| < cut off (<120g/L female, <130g/L male) |  | 31 (47.7) |  |  |
| Albumin | 65 |  | 33.6 (3.8) | 23-42 |
| < 35g/L |  | 38 (58.5) |  |  |
| HbA1c | 35^◊^ |  | 6.6 (1.4) | 4.0-9.8 |
| > 6.5% |  | 15 (42.9) |  |  |
| Missing data | 30 |  |  |  |
| Folate | 43^◊^ |  | 29.4 (10.9) | 7.9-52.9 |
| > 6nmol/L |  | 43 (100) |  |  |
| Missing data | 22 |  |  |  |
| B12 | 45^◊^ |  | 291.0 (161.0-292.0)^†^ | 90-640 |
| ≤ 200pmol/L |  | 24 (53.3) |  |  |
| Missing data | 20 |  |  |  |
| Iron Level (umol/L) | 38^◊^ |  | 8.5 (6.0-12.3)^†^ | 2-30 |
| Missing data | 27 |  |  |  |
| Ferritin (ug/L) | 39^◊^ |  | 68.0 (34.0-229.0)^†^ | 14-584 |
| ≤ cut off (<11 female, <24 male) |  | 4 (10.3) |  |  |
| Missing data | 26 |  |  |  |
| Hospital admissions for same pressure injury | 84 | 59 (70.2) |  |  |
| Total days in hospital for same PI | 50^◊^ |  | 26.5 (14.0-85.8)^†^ | 1-390 |
| Missing data | 9 |  |  |  |

^◊^ Variable had some missing data, therefore results are reflective of available data. *^a^ Body Mass Index (BMI) categories for people with SCI/D: underweight ≤ 15.99; Healthy weight = 16.00 – 21.99; Overweight or obese ≥ 22.00.* ^†^ Median (IQR).

Supplementary Table 5. Frequency and nature of hospital-acquired pressure injuries following flap surgery (*n* = 84)

| **Variable** | ***n*** | **Frequency** | **%** |
| --- | --- | --- | --- |
| Number of hospital-acquired PIs | 84 |  |  |
| 0 |  | 63 | 75.0 |
| 1 |  | 13 | 15.5 |
| 2 |  | 5 | 6.0 |
| 3-4 |  | 3 | 3.6 |
| PI stage | 33 |  |  |
| 1 |  | 9 | 27.3 |
| 2 |  | 12 | 36.4 |
| 3 |  | 2 | 6.1 |
| 4 |  | 2 | 6.1 |
| Suspected Deep Tissue Injury |  | 1 | 3.0 |
| Unknown |  | 7 | 21.2 |
| PI location | 33 |  |  |
| Ischial tuberosity (incl perineum) |  | 5 | 15.2 |
| Greater trochanter |  | 11 | 33.3 |
| Sacrum (incl. sacral and sacrococcygeal) |  | 4 | 12.1 |
| Foot (incl. ankle, foot, malleolus, heel, toe, calcaneal) |  | 5 | 15.2 |
| Other lower limb (thigh/knee) |  | 3 | 9.1 |
| Other |  | 5 | 15.2 |
| PI cause | 33 |  |  |
| Strict bedrest / positioning |  | 14 | 42.4 |
| Poor adherence to pressure area cares |  | 4 | 12.1 |
| Systemic illness (e.g., generally unwell, febrile episodes, altered consciousness) |  | 6 | 18.2 |
| Equipment, aid, or appliance-related |  | 5 | 15.2 |
| Catheter injury |  | 2 | 6.1 |
| Other |  | 7 | 21.2 |

*PI = pressure injury.*

Supplementary Table 6. Mann-Whitney U results.

| **Risk factors** | **Complications** | ***n*** | **Mean Rank** | **U** | **Z** | ***p*** | **r (effect size)** |
| --- | --- | --- | --- | --- | --- | --- | --- |
| Myocutaneous | Local | No = 44 | 28.97 | 284.50 | -1.67 | .107 | -0.21 small |
|  |  | Yes = 17 | 36.26 |  |  |  |  |
|  | General | No = 44 | 31.36 | 358.00 | -0.26 | .798 | -0.03 |
|  |  | Yes = 17 | 30.06 |  |  |  |  |
|  | Total | No = 44 | 30.97 | 372.00 | -0.02 | .983 | -0.00 |
|  |  | Yes = 17 | 31.09 |  |  |  |  |
| Osteomyelitis | Local | No = 53 | 29.11 | 112.00 | -2.47 | .019* | -0.32 medium |
|  |  | Yes = 8 | 43.50 |  |  |  |  |
|  | General | No = 53 | 31.68 | 176.00 | -0.78 | .447 | -0.10 small |
|  |  | Yes = 8 | 26.50 |  |  |  |  |
|  | Total | No = 53 | 30.69 | 195.50 | -0.36 | .728 | -0.05 |
|  |  | Yes = 8 | 33.06 |  |  |  |  |
| Current smoker | Local | No = 44 | 31.19 | 365.50 | -0.16 | .953 | -0.02 |
|  |  | Yes = 17 | 30.50 |  |  |  |  |
|  | General | No = 44 | 33.40 | 268.50 | -1.73 | .085 | -0.22 small |
|  |  | Yes = 17 | 24.79 |  |  |  |  |
|  | Total | No = 44 | 33.43 | 267.00 | -1.74 | .082 | -0.22 small |
|  |  | Yes = 17 | 24.71 |  |  |  |  |
| Spasticity on admission | Local | No = 52 | 29.20 | 140.50 | -2.20 | .038* | -0.28 small |
|  |  | Yes = 9 | 41.39 |  |  |  |  |
|  | General | No = 52 | 29.68 | 165.50 | -1.42 | .161 | -0.18 small |
|  |  | Yes = 9 | 38.61 |  |  |  |  |
|  | Total | No = 52 | 29.38 | 150.00 | -1.73 | .085 | -0.22 small |
|  |  | Yes = 9 | 40.33 |  |  |  |  |
| Kyphoscoliosis / pelvic obliquity | Local | No = 14 | 22.71 | 179.00 | -0.52 | .794 | -0.08 |
|  |  | Yes = 28 | 20.89 |  |  |  |  |
|  | General | No = 14 | 16.82 | 130.50 | -1.78 | .076 | -0.27 small |
|  |  | Yes = 28 | 23.84 |  |  |  |  |
|  | Total | No = 14 | 17.39 | 138.50 | -1.57 | .120 | -0.27 small |
|  |  | Yes = 28 | 23.55 |  |  |  |  |
| Tetraplegia | Local | No = 38 | 31.14 | 431.50 | -0.10 | .925 | -0.01 |
|  |  | Yes = 23 | 30.76 |  |  |  |  |
|  | General | No = 38 | 30.25 | 408.50 | -0.43 | .666 | -0.06 |
|  |  | Yes = 23 | 32.24 |  |  |  |  |
|  | Total | No = 38 | 30.25 | 408.50 | -0.43 | .667 | -0.06 |
|  |  | Yes = 23 | 32.24 |  |  |  |  |
| More than 1 PI on admission | Local | No = 31 | 23.39 | 229.00 | -3.94 | <.001*** | -0.50 large |
|  |  | Yes = 30 | 38.87 |  |  |  |  |
|  | General | No = 31 | 31.06 | 463.00 | -0.03 | .977 | -0.00 |
|  |  | Yes = 30 | 30.93 |  |  |  |  |
|  | Total | No = 31 | 28.27 | 380.50 | -1.24 | .217 | -0.16 small |
|  |  | Yes = 30 | 33.82 |  |  |  |  |
| Increased pain | Local | No = 33 | 26.05 | 298.50 | -2.74 | .006** | -0.35 medium |
|  |  | Yes = 28 | 36.84 |  |  |  |  |
|  | General | No = 33 | 30.86 | 457.50 | -1.07 | .947 | -0.14 small |
|  |  | Yes = 28 | 31.16 |  |  |  |  |
|  | Total | No = 33 | 28.91 | 393.00 | -1.01 | .311 | -0.13 small |
|  |  | Yes = 28 | 33.46 |  |  |  |  |
| Previous flap surgeries | Local | No = 39 | 26.73 | 262.50 | -2.89 | .004** | -0.37 medium |
|  |  | Yes = 22 | 38.57 |  |  |  |  |
|  | General | No = 39 | 32.99 | 351.50 | -1.19 | .236 | -0.15 small |
|  |  | Yes = 22 | 27.48 |  |  |  |  |
|  | Total | No = 39 | 31.21 | 421.00 | -0.12 | .903 | -0.02 |
|  |  | Yes = 22 | 30.64 |  |  |  |  |
| Urinary incontinence impacting on flap site | Local | No = 48 | 29.48 | 239.00 | -1.49 | .157 | -0.19 small |
|  |  | Yes = 13 | 36.62 |  |  |  |  |
|  | General | No = 48 | 29.45 | 237.50 | -1.34 | .185 | -0.17 small |
|  |  | Yes = 13 | 36.73 |  |  |  |  |
|  | Total | No = 48 | 28.60 | 197.00 | -2.05 | .040* | -0.26 small |
|  |  | Yes = 13 | 39.85 |  |  |  |  |
| Mental health | Local | No = 35 | 30.50 | 437.50 | -0.30 | .768 | -0.04 |
|  |  | Yes = 26 | 31.67 |  |  |  |  |
|  | General | No = 35 | 32.63 | 398.00 | -0.85 | .397 | -0.11 small |
|  |  | Yes = 26 | 28.81 |  |  |  |  |
|  | Total | No = 35 | 31.66 | 432.00 | -0.34 | .734 | -0.05 |
|  |  | Yes = 26 | 30.12 |  |  |  |  |
| Traumatic Brain Injury | Local | No = 50 | 30.50 | 250.00 | -0.54 | .614 | -0.07 |
|  |  | Yes = 11 | 33.27 |  |  |  |  |
|  | General | No = 50 | 32.03 | 223.50 | -0.98 | .334 | -0.13 small |
|  |  | Yes = 11 | 26.32 |  |  |  |  |
|  | Total | No = 50 | 31.50 | 250.00 | -0.48 | .643 | -0.06 |
|  |  | Yes = 11 | 28.73 |  |  |  |  |

Note. Exact sig. reported when groups have fewer than 20 in each group and asymp. sig reported when groups have <20 in each group. **p* < .05, ***p*<.01, ****p*<.001
